# Methylomes in Vegans versus Pescatarians and Nonvegetarians

**DOI:** 10.3390/epigenomes4040028

**Published:** 2020-12-11

**Authors:** Valery Filippov, Karen Jaceldo-Siegl, Alexey Eroshkin, Vasiliy Loskutov, Xin Chen, Charles Wang, Penelope J. Duerksen-Hughes

**Affiliations:** 1 Department of Basic Sciences, School of Medicine, Loma Linda University, Loma Linda, CA 92354, USA;; 2 Department of Nutrition, School of Public Health, Loma Linda University, Loma Linda, CA 92354, USA;; 3 Sanford Burnham Prebys Medical Discovery Institute, La Jolla, CA 92037, USA;

**Keywords:** differential DNA methylation, epigenetics, pathway analysis, vegan diet

## Abstract

Epigenetic studies in animal models have demonstrated that diet affects gene regulation by altering methylation patterns. We interrogated methylomes in humans who have different sources of protein in their diet. We compared methylation of DNA isolated from buffy coat in 38 vegans, 41 pescatarians and 68 nonvegetarians. Methylation data were obtained using Infinium HumanMethylation450 arrays and analyzed using the Partek Genomic software. Differences in differentially methylated sites were small, though with the use of relaxed statistical tests we did identify diet-associated differences. To further test the validity of these observations, we performed separate and independent comparisons of the methylation differences between vegans and nonvegetarians, and between vegans and pescatarians. The detected differences were then examined to determine if they were enriched in specific pathways. Pathway analysis revealed enrichment of several specific processes, including homeobox transcription and glutamate transport. The detected differences in DNA methylation patterns between vegans, pescatarians, and nonvegetarians enabled us to identify 77 CpG sites that may be sensitive to diet and/or lifestyle, though high levels of individual-specific differences were also noted.

## 1. Introduction

Vegetarianism, a diet characterized by abstinence from meats, poultry, and fish, has been reported to reduce risk factors for several chronic diseases, and, in particular, is associated with a lower incidence of and mortality from ischemic heart disease [1]. A recent systematic review and meta-analysis of observational analyses further provide evidence of the benefits of vegan diets (abstention from all flesh foods, dairy, and eggs) on cancer [2]. The inclusion of a healthy vegetarian diet in the 2015–2020 Dietary Guidelines for Americans [3] likely will increase vegetarianism as a dietary choice among all ages. Nevertheless, the processes and mechanisms that explain how diet may interact with gene regulation, epigenetics included, are largely unknown, though further understanding of these factors has the potential to significantly improve health and quality of life [4].

Recent advances regarding the association of diet and epigenetic changes have been summarized in a review by Sapienza and Issa [5]. Significant advances have been achieved in our understanding of the role of dietary components on epigenetic alterations using animal models. For example, it has been shown that both maternal methyl-donor supplementation and calorie restriction induces epigenetic changes in murine offspring [6,7], and that a maternal high fat diet affects the methylomes of neonatal offspring rats [8]. Studies on human subjects are much less available, largely due to difficulties in the collection of sufficient and appropriate samples, and often display inconsistent results [5]. However, epidemiological human studies have identified dietary factors that may explain epigenetic alterations, possibly including a transgenerational effect that depends on maternal food consumption [9]. Direct evidence of such dietary regulation of epigenetic changes was found by studying methylation patterns of several loci in children of individuals who were exposed to famine during World War II in the Netherlands [10].

In this present study, we applied comparative methylome analysis to characterize and compare the DNA methylation patterns in vegans, pescatarians and nonvegetarians. We then employed pathway analysis to assess whether there was gene enrichment in specific processes, as this would indicate nonrandom selection of genes. Performing these analyses independently for both the vegan/pescatarian and the vegan/nonvegetarian comparisons enabled us to strengthen our conclusion that diet can influence epigenetic patterns.

## 2. Materials and Methods

### 2.1. Subjects and Study Design

The Adventist Health Study-2 (AHS-2) is a prospective cohort of 96,592 Adventists in North America established between 2002 and 2007. Recruitment and selection methods have been reported previously in detail [11]. This study was performed by protocol #5130319 approved by the Institutional Review Board of Loma Linda University and all participants gave written consent at enrollment. From this collection, we selected a sample of 147 individuals with contrasting dietary patterns (vegan, pescatarian, and nonvegetarian), matched by gender and age, for these methylation studies: 38 vegans, 41 pescatarians and 68 nonvegetarians (omnivores).

### 2.2. Dietary Assessment

Nutrient and food intake were assessed by a previously validated food frequency questionnaire[12]. Dietary intake estimates were calculated using the product-sum method [13] where intake = sum[(weighted frequency of use of a food) × (weighted portion size consumed of that food) × (nutrient content in a standard serving size of that food)]. Dietary patterns were determined according to the reported frequency of intake of animal-based foods from the FFQ [14]. Specifically, vegans consumed eggs/dairy, fish, and all other meats never or rarely; lacto-ovo vegetarians consumed eggs/dairy 1 time/mo or more, but fish and all other meats less than 1 time/mo; pescatarians consumed fish 1 time/mo or more, but all other meats less than 1 time/mo; semi vegetarians consumed non-fish meats 1 time/mo or more, and all meats combined (fish included) 1 time/mo or more but no more than 1 time/wk; and last, nonvegetarians consumed non-fish meats 1 time/mo or more, and all meats combined (fish included) more than 1 time/wk.

### 2.3. Anthropometric and Lifestyle Measures

We measured body weight and height using standard protocols, and collected fasting blood samples at field clinics held in church halls. A questionnaire completed at baseline provided information on physical activity (min/week), sleep hours (h/day), perception of one’s own health (excellent, good, fair), prevalent cancer and CVD comorbidities, and cigarette smoking (never or ever).

### 2.4. Blood Collection

Fasting blood was obtained at field clinics, collected in heparin tubes, and shipped overnight in insulated thermal containers packed with frozen icepacks to the processing laboratory at Loma Linda, CA. Buffy coat was removed and diluted to a final volume of 8 mL with phosphate buffered saline, then aliquoted into straws and frozen at −180 °C in nitrogen vapor.

### 2.5. DNA Isolation and DNA Methylation Analysis

Genomic DNA from buffy coat samples was isolated using the Quick-gDNA MiniPrep kit (Zymo Research, Irvine, CA, USA) according to the manufacturer’s protocol. Genome-wide methylation analysis was carried out using the Illumina Infinium HumanMethylation450 BeadChip platform at the UCLA Neuroscience Genomics Core in two separate batches. Raw data was summarized into BeadStudio IDAT files for further analysis.

### 2.6. Methylation Data Analysis

**Figure 1.**
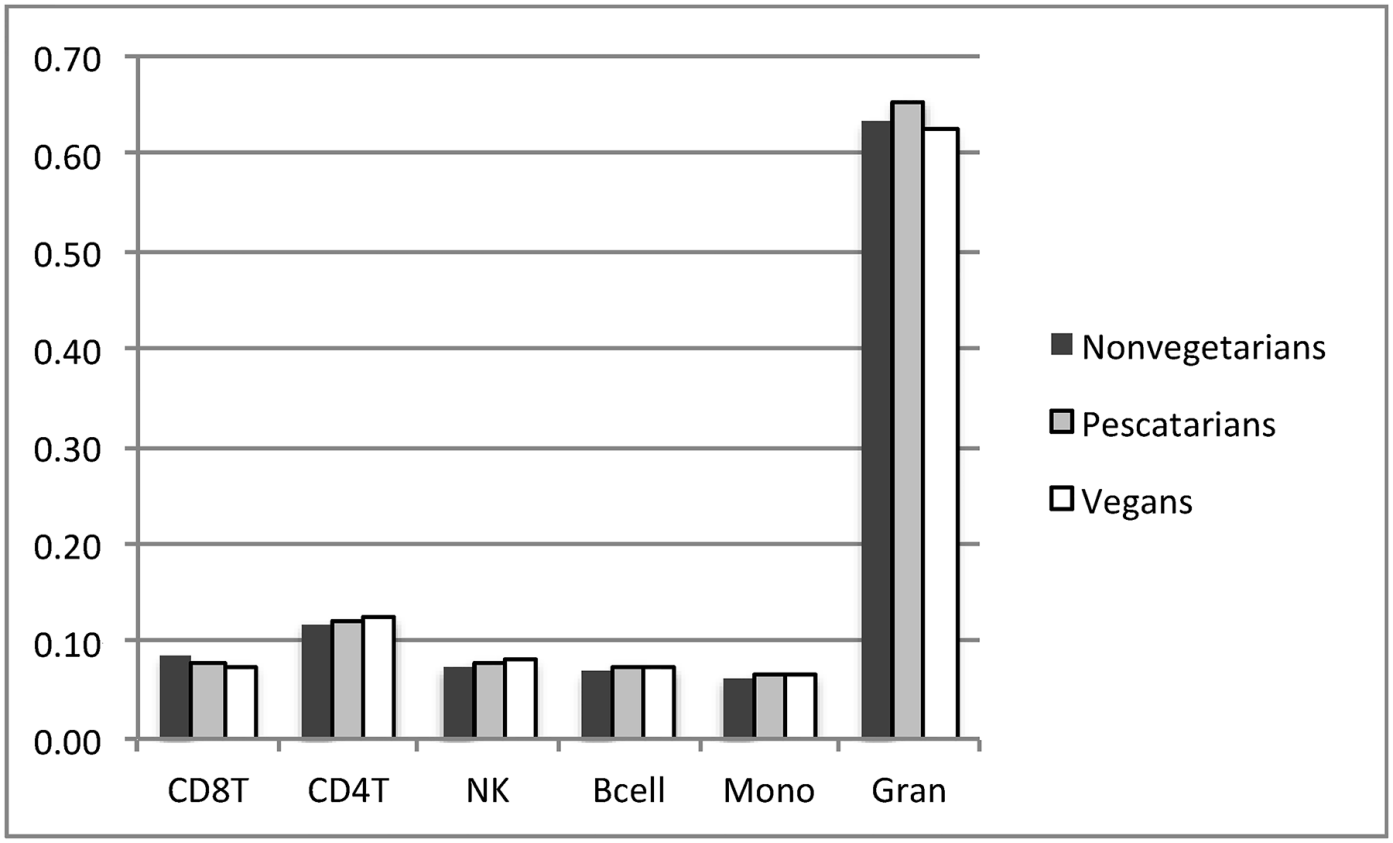
Composition of cell types in blood samples estimated by Houseman method using R minfi package.

Methylation data, based on methylation beta values, were analyzed using the Partek Genomic Suite (Partek, St. Lois, MO, USA). Comparative analyses were carried out separately between vegans and nonvegetarians and between vegans and pescatarians. Batch correction was carried out using the ANOVA-based batch correction method embedded within the commercial program Partek. We also used the R minfi package, based on Houseman’s method to estimate blood cell heterogeneity in buffy coat samples [15], with the analysis indicating no significant variation between cell types between the three groups as shown on Figure 1.

Raw data was normalized using the SWAN (Subset-quantile With Array Normalization) method. Normalized data were subjected to quality control analysis, and nine samples with distorted signal frequency histograms were culled out. Primary normalization was followed by batch effect correction and quantile normalization. Data from probes representing CpG sites from chromosomes X and Y and those close to known SNPs (±10 nucleotides) were removed from further analysis. Data from the remaining probes were analyzed to identify sites displaying differential CpG methylation between the three diets. Initial filtering using an FDR < 0.05 cutoff for nonvegetarians revealed only one site, consistent with the expected modest differences due to diet. This CpG site was also the only one to appear with FDR cutoffs of 0.1, 0.2, and 0.3. Subsequently, relaxed conditions of an ANOVA procedure with a Fold Change cutoff = 1.2 (20% difference) and a *p*-value cutoff = 0.05 were employed to detect differentially methylated sites. Differentially methylated genes identified by this explorative approach, which does not correct for multiple testing, were then subjected to pathway analysis.

### 2.7. Biological Pathway Analyses

To analyze in more detail the potential biological effects of a vegan diet due to variations in methylation patterns, we conducted biological pathway analyses using the Ingenuity Pathway Analysis (IPA) web-based analysis tool, as well as DAVID Bioinformatics Resources [16]. These analyses allowed us to statistically evaluate if the CpG differentially methylated sites we identified were specifically enriched with genes that are engaged in certain pathways, as well as any possible crosstalk between them. The original file generated by analysis in the Partek suite, containing the name of each gene, fold change, and P value information was uploaded into the IPA system. Using a cut-off value of 1.25 for fold change, a new dataset was produced, which was used for Core Analysis to identify relevant canonical pathways. Pathways with P values (Fisher’s test) of less than 0.05 were considered significant. The same list of genes was analyzed with DAVID tools to identify enriched functional-related gene groups.

## 3. Results

### 3.1. Characterization of Study Subjects

We conducted three group comparisons using the Student’s *t*–test or the Fisher’s exact test as appropriate: Vegan vs. nonvegetarian, vegan vs. pescatarian, and pescatarian vs. nonvegetarian. Comparisons of selected characteristics of the study sample are shown in Table 1.

Compared to vegans, BMI was higher among pescatarians (*p* = 0.05) and nonvegetarians (*p* < 0.0001), and pescatarians had lower BMI than nonvegetarians (*p* = 0.02). Exercise duration (min/week) was lower among pescatarians (*p* = 0.002) and nonvegetarians (*p* = 0.0001) compared to vegans, but no significant difference was observed between pescatarians and nonvegetarians. Prevalence of cancer, CVD comorbidities, and health perception were not significantly different among the comparison groups. Adventists notably are a non-smoking population, and in this sample < 3% of vegans, 20% pescatarians, and <15% of non-vegetarians ever smoked. Compared to vegans (67.8 y), average age was not significantly different among pescatarians (69.9 y) or nonvegetarians (66.4 y). However, pescatarians were older than nonvegetarians (*p* = 0.02) (Table 1).

Table 1 also shows dietary intake characteristics of our study subjects. Compared to vegans, carbohydrate intake was lower among nonvegetarians (*p* < 0.0001) and pescatarians (*p* = 0.004), and fat intake was higher among nonvegetarians (*p* < 0.0001) and pescatarians (*p* = 0.007). Though energy and total protein intake (as % of energy) did not differ between the comparison groups, we found significant trends in the intake of vegetable protein and animal protein (as % of protein). Not surprisingly, vegetable protein was highest among vegans, intermediate among pescatarians and lowest among nonvegetarians, and animal protein intake was highest among nonvegetarians, followed by pescatarians and negligible amounts among vegans (*p* < 0.0001 for all comparison groups). Among the essential amino acids (where the source must come from the diet), intake tended to be highest among nonvegetarians, intermediate among pescatarians, and lowest among vegans (*p*-values ≤ 0.05 for nearly all comparison groups), with the exceptions of tryptophan, threonine, and phenylalanine. Among the non-essential amino acids, we found similar trends for the intake of tyrosine and proline; however, intake of glutamate, aspartate, arginine, glycine, and serine tended to be highest among vegans, then pescatarians, and lowest among nonvegetarians (*p*-values ≤ 0.05 for nearly all comparison groups). Among the nutrients involved in one-carbon metabolism, dietary intake of vitamin B2, vitamin B6, folate, and betaine were not significantly different between the comparison groups. Vitamin B12 was significantly higher in pescatarians compared to vegans (*p* = 0.03), and compared to nonvegetarians, choline intake was lower (*p* = 0.0002) among vegans and pescatarians (*p* = 0.01).

### 3.2. Identification of Differential Methylation Sites

Methylation data was obtained and analyzed using the Infinium HumanMethylation450 beadchip array and Partek Genomic Suite software. Initial comparison of methylation profiles between vegans and nonvegetarians using the following statistical parameters: FDR (False Discovery Rate) < 0.05 and fold change difference more than 1.2 or less than −1.2, showed that no CpG sites passed these criteria. The one site that did meet the FDR criteria (within the OR1M1 olfactory receptor gene) did not meet the fold change criteria, and was therefore excluded from further analysis. For the comparison between vegans and pescatarians, no sites met the FDR criteria.

We then used relaxed statistical conditions by using unadjusted *p*-values < 0.05 and a Fold-Change (FC) cutoff of 1.2, we found 523 CpG sites that passed these criteria when we compared vegan vs. nonvegetarians, of which 424 were located within gene areas (Table S1). We then performed the same analysis to compare methylation data between the same vegan group and the pescatarians. This led to the detection of 358 CpG sites, of which 271 were located within known gene areas (Table S2). A comparison of the differentially methylated CpG sites between nonvegetarians and pescatarians revealed that 77 sites are common for both these diets, and that 55 of them are within known genes (Table S3).

To further explore whether the inclusion of animal-derived proteins in the diet may be reflected in epigenetic differences, we compared the lists of the ten top genes displaying differentially methylated CpG sites that showed the highest differences in methylation status for nonvegetarians and pescatarians (Tables 2 and 3, correspondingly).

Three genes that appeared in the results from both analyses are highlighted in Tables 2 and 3, indicating their highly methylated status in vegans as compared to both pescatarians and nonvegetarians.

A gene list created by comparing the differentially methylated CpG sites observed in nonvegetarians and vegans was used to perform DAVID functional annotation clustering using its own Knowledgebase. This analysis revealed that out of 49 identified functional groups of genes (Table S4), the top cluster, as shown in Table 4, demonstrates enrichment of homeobox transcriptional factors. This enrichment is significant by a variety of statistical tests. The potential of these transcription factors to amplify differential activation may indicate that the presence or absence of animal proteins in the diet may lead to much larger differences in gene expression regulation than the relatively modest fold-changes detected by an epigenetics approach might suggest.

Similar results were obtained when we performed the same analysis under the same conditions using the gene list based on differential methylation detected in pescatarians vs. vegans. Table 5 shows that homeobox transcription factors again form the top functional cluster among all the genes used in analysis. However, statistical characteristics for pescatarians are weaker (Table 5).

It is important to note that, out of 8 genes encoding homeobox transcription factors detected in pescatarians, 3 are found also in nonvegetarians, which indicate on nonrandom differences in methylation patterns (Table 6).

### 3.3. Pathway Analysis

To analyze in more detail the potential biological effects of a vegan diet due to variations in methylation patterns, we employed the DAVID (Database for Annotation, Visualization and Integrated Discovery) online resource [16]. This resource uses several pathway databases, including the KEGG (Kyoto Encyclopedia of Genes and Genomes) database, which contains pathway maps. Such analyses are useful in evaluating the biological relevance of high-throughput data, particularly when relaxed statistics have been used. In particular, pathway analysis allowed us to statistically evaluate whether the group of identified genes is specifically enriched with genes that are engaged in certain pathways/processes.

The pathway analysis of pescatarians vs. vegans found only 3 processes that were enriched in this group of genes (Table 7). However, two of these processes were also found in the vegan vs. nonvegetarian analysis (Table 8): The Hippo signaling pathway and the Glutamatergic Synapse pathway. These two pathways are highlighted in table for pescatarians (Table 7).

Table 8 shows 6 top pathways detected among the genes with differential methylation noted in nonvegetarians. Although only 2 of these pathways are the same as those noted for pescatarians, some of the pathways share overlapping sets of genes and this can lead to some redundancy. For example, the four pathways marked in grey in Table 8 share genes involved in processes linked to GABA regulation and thus form one cluster.

## 4. Discussion

We performed comparative DNA methylation analyses of DNA isolated from the blood cells of vegans versus nonvegetarians and of vegans versus pescatarians using the Illumina Infinium 450k human assay. Importantly, in this study, DNA methylation analyses of vegans against individuals with similar but not identical diets containing animal-derived sources of protein led to the identification of genes clustered in similar but not completely identical functional groups.

Identification and interpretation of DNA methylation differences associated with diet preferences in humans are complicated by several interdependent aspects. The fact that humans are the study subjects brings significant biological variability to genotype-environment (GxE) interactions, leading to inter- and intra-individual (i.e., over time) methylome variations in humans [17]. Another factor relevant to this study is the likelihood that vegans in general tend to be more aware of the need for a healthy lifestyle, leading to differences in BMI and physical activity between our two subgroups. These differences in BMI and physical activity have the potential to contribute to our observed differences in methylation patterns. Unfortunately, it is not possible currently to dissect out the differential impacts from these factors due to the limited numbers of subjects in this study. Therefore, it may be appropriate to interpret our findings in the context of the broader emphasis on a healthy lifestyle exhibited by vegans.

We also note that there are multiple mechanisms through which protein expression and metabolism—and thereby, health outcomes—can be modulated, of which epigenetic changes are only one. Modest changes in gene expression have been shown in studies of Prudent and Western dietary patterns in healthy individuals [18]. Therefore, we expected to find that any epigenetic changes related to diet preferences, especially in the DNA of blood cells, should also be modest, if indeed, they could be detected. As noted above, pathway analysis statistically evaluates all observed changes in activation or inactivation of proteins engaged in a particular pathway. The fact that pathway analysis did detect differences in the modulation of specific pathways, even though the only input was changes in methylation status, is noteworthy.

All of these factors contribute to the modest ability of these types of analyses to detect the full extent of gene expression changes that could be related to diet. To address these challenges and to increase the likelihood of detecting genuine associations of methylome changes with diet preferences, relaxed criteria were applied. These relaxed criteria increase the likelihood of detecting of both true and false positives. A rough estimation regarding the relative numbers of true and false positives can be suggested from analysis of the top ten most differentially methylated genes (Tables 2 and 3), where 3 genes, or approximately 30% of this top grouping, were identified in both analyses. Another independent layer of analysis designed to increase the probability of detecting true differences was to search for identical sites (genes) found to be differentially methylated in DNA from individuals who follow either of two closely related but not identical diets, where these genes are involved in well-known biological processes/pathways. That is, identification of changes in the methylation of the same genes and pathways in individuals who consume no animal proteins as compared to those who restrict their diet to only fish, and as compared to those who are to full omnivores, strengthens the conclusions we can draw.

To our knowledge, this is the first large-scale attempt to apply an epigenome-wide approach to examine the relationship between diet—and in particular, a diet restricted to plant-based food-and methylome changes. In general, the magnitude of individual changes was relatively modest—we used a cut-off of 1.2 for the Partek analysis. Such changes are in line with what has been previously reported regarding the relationship between methylation levels and nutrition. For example, fortification of food with the methyl donor, folic acid, increased the methylation level of a DM site within the IGF2 gene in human blood cells by 4.5%, a value that is believed to be comparable to results obtained in other mammals [19].

Genome-wide methylation analysis of 147 individuals, 38 of which are vegans, allowed us to obtain sufficient data to gain biologically interesting results for epigenetic differences in methylation in vegans versus pescatarians and in vegans versus nonvegetarians. Interestingly, we found that vegans had a higher methylation status in the majority of the differentially methylated sites, with DNA hypomethylation occurring in only 4% of all DM probes in the nonvegetarian comparison; this value was 33% for the pescatarian comparison. This is consistent with a study comparing methylation status between two empirically-defined dietary patterns, where a prudent diet (characterized by high intakes of fruits and vegetables) compared to a Western diet (characterized by high intake of grains, potatoes, meats and oils) was associated with less DNA hypomethylation [20].

One of the most intriguing outcome of our analysis was the simultaneously increased methylation of regulatory elements of genes involved in transport of two major neurotransmitters: GABA (inhibitory), and glutamate (excitatory) in nonvegetarians. Interestingly, microbiome work by David et al. also found altered expression of genes involved in amino acid metabolism (GABA and glutamate) and distinct enrichment patterns associated with animal- and plant-based diets [21]. This finding may be partially explained by the amino acid composition of the vegan diet, as a number of important products are synthesized from amino acids.

Our finding that transcriptional factors, and in particular, homeobox regulators, were enriched in the list of differentially methylated genes (Tables 4–6), raises the question of how many genes may have been affected by these diet restrictions. This is also true for the HIPPO pathway, where differential methylation of several genes, as well as pathway enrichment, were found in both non-vegan diets. HIPPO pathway plays an important role in the regulation of tissue homeostasis [22]. Direct investigation regarding the expression of these genes would be quite useful in efforts to further understand the potential role of diet in health and disease development.

## 5. Conclusions

We performed genome-wide comparisons of methylation patterns in the DNA of blood cells isolated from vegans, pescatarians and nonvegetarians. The results allowed us to identify CpG sites that may display differential DNA methylation. We also found that the set of genes with differentially methylated CpG sites in vegans are enriched in specific common functional clusters/pathways, when compared with DNA isolated from either pescatarians or nonvegetarians. This suggests that these epigenetic changes are diet-related and have the potential to control specific physiological processes. Future efforts focused on direct investigation of gene expression will continue to expand our understanding of the influence of nutrition on major biological processes that regulate health and quality of life.

## Supplementary Material

Supplementary

## Figures and Tables

**Table 1.** Selected characteristics of study sample comparing vegans, pesco vegetarians, and omnivores.

Demographic and Lifestyle	*n* = 38	*n* = 41	*n* = 68	Vegan vs. Omni	Pesco vs. Omni	Vegan vs. Pesco
Males (%)						
Age at collection, mean ± SD (years)	52.63	48.78	47.06	0.58	0.86	0.73
BMI at collection, mean ± SD	67.78 (8.50)	69.93 (7.87)	66.42 (6.73)	0.37	0.02	0.25
Exercise, mean ± SD (min/week)	23.62 (4.23)	25.36 (3.63)	27.07 (3.69)	<0.0001	0.02	0.05
Sleep hours, mean ± SD (hrs/day)	152.93 (109.59)	76.68 (86.64)	69.23 (95.72)	0.0001	0.58	0.002
Perception of one’s own health (%)	7.30 (0.81)	7.03 (0.95)	6.94 (0.93)	0.05	0.65	0.18
Excellent				0.07	0.82	0.22
Good	25.71	22.50	17.65			
Fair	54.29	70.00	75.00			
Prevalent cancer (%)	20.00	7.50	7.35			
Yes				0.57	0.66	0.91
No	10.53	9.76	7.35			
CVD comorbidities (%)	89.47	90.24	92.65			
None				0.35	0.74	0.57
1 or more	76.32	70.73	67.65			
Cigarette smoke (%)	23.68	29.27	32.35			
Never				0.05	0.51	0.02
Ever	97.37	80.49	85.29			
	2.63	19.51	14.71			
**Dietary intake, mean** ± **SD**						
Energy (kcal/d)						
Carbohydrate (% of kcal)	1769.82 (585.13)	2029.3 (609.7)	1927.77 (667.22)	0.23	0.43	0.06
Fat (% of kcal)	57.58 (6.96)	52.83 (7.17)	49.74 (8.13)	<0.0001	0.05	0.004
Protein (% of kcal)	28.72 (6.92)	33.20 (7.29)	35.66 (7.85)	<0.0001	0.11	0.007
Vegetable protein (% of protein)	13.70 (2.80)	13.97 (2.42)	14.61 (2.21)	0.071	0.07	0.65
Animal protein (% of protein)	96.87 (2.57)	80.39 (12.56)	60.63 (14.82)	<0.0001	<0.0001	<0.0001
*Essential amino acids*	3.89 (2.33)	19.92 (12.41)	39.41 (14.75)	<0.0001	<0.0001	<0.0001
Leucine (% of protein)						
Valine (% of protein)	6.98 (0.48)	7.40 (0.35)	7.61 (0.36)	<0.0001	0.003	<0.0001
Isoleucine (% of protein)	4.66 (0.25)	4.89 (0.21)	4.98 (0.21)	<0.0001	0.03	<0.0001
Histidine (% of protein)	3.95 (0.24)	4.12 (0.20)	4.29 (0.19)	<0.0001	<0.0001	0.0007
Methionine (% of protein)	2.42 (0.13)	2.45 (0.08)	2.54 (0.1)	<0.0001	<0.0001	0.24
Tryptophan (% of protein)	1.50 (0.12)	1.73 (0.17)	1.92 (0.16)	<0.0001	<0.0001	<0.0001
Threonine (% of protein)	1.26 (0.07)	1.23 (0.06)	1.22 (0.06)	0.002	0.16	0.1
Lysine (% of protein)	3.5 (0.26)	3.47 (0.20)	3.55 (0.17)	0.33	0.04	0.54
Phenylalanine (% of protein)	4.47 (0.48)	4.93 (0.48)	5.55 (0.64)	<0.0001	<0.0001	<0.0001
*Non-essential amino acids*	4.88 (0.24)	4.88 (0.11)	4.75 (0.16)	0.005	<0.0001	0.99
Glutamate (% of protein)						
Aspartate (% of protein)	23.07 (1.94)	22.82 (1.58)	22.18 (1.64)	0.010	0.05	0.53
Alanine (% of protein)	10.20 (1.33)	9.50 (0.79)	9.07 (0.71)	<0.0001	0.004	0.005
Cysteine (% of protein)	4.40 (0.38)	4.25 (0.27)	4.29 (0.23)	0.06	0.46	0.05
Arginine (% of protein)	1.61 (0.14)	1.65 (0.16)	1.65 (0.16)	0.21	0.95	0.28
Tyrosine (% of protein)	6.79 (0.79)	6.12 (0.73)	5.61 (0.66)	<0.0001	0.0003	0.0002
Glycine (% of protein)	3.04 (0.28)	3.24 (0.25)	3.38 (0.20)	<0.0001	0.002	0.001
Proline (% of protein)	4.27 (0.31)	3.92 (0.34)	3.79 (0.32)	<0.0001	0.05	<0.0001
Serine (% of protein)	6.65 (0.92)	7.24 (0.86)	7.31 (0.73)	<0.0001	0.64	0.004
*Micronutrients*	4.87 (0.29)	4.95 (0.16)	4.79 (0.19)	0.10	<0.0001	0.12
Vitamin B2, mg/1000 kcal						
Vitamin B6, mg/1000 kcal	2.85 (3.28)	3.64 (5.18)	2.78 (6.62)	0.95	0.43	0.42
Vitamin B-12, mcg/1000 kcal	3.56 (7.04)	8.54 (15.42)	5.14 (10.21)	0.4	0.17	0.07
Folate, mcg/1000 kcal	3.93 (4.43)	7.64 (9.38)	6.19 (11.23)	0.24	0.49	0.03
Choline, mg/1000 kcal	460.0 (387.8)	440.1 (193.0)	401.9 (214.1)	0.19	0.35	0.67
Betaine, mg/1000 kcal	117.0 (21.73)	122.9 (29.40)	136.9 (27.61)	0.0002	0.01	0.32
					0.91	0.6

**Table 2.** Ten top genes wittabh the greatest differential CpG methylation status in vegans vs. nonvegetarians.

Entrez Gene Name	Symbol	Fold Change	Location	Type(s)
mitochondrial ribosomal protein L19	MRPL19	2.517	Cytoplasm	other
proline rich 7, synaptic	PRR7	2.071	Other	other
glutathione S-transferase C-terminal domain containing	GSTCD	1.879	Cytoplasm	enzyme
chromosome 7 open reading frame 50	C7orf50	1.743	Other	other
dynein axonemal heavy chain 10	DNAH10	1.636	Cytoplasm	other
solute carrier family 38 member 6	SLC38A6	1.619	Plasma Membrane	transporter
glutathione S-transferase theta 1	GSTT1	1.594	Cytoplasm	enzyme
calcium voltage-gated channel auxiliary subunit beta 2	CACNB2	1.541	Plasma Membrane	ion channel
family with sequence similarity 19 member A5, C-C motif chemokine like	FAM19A5	1.524	Extracellular Space	other
transmembrane protein 229A	TMEM229A	1.523	Other	other

Genes highlighted in grey are common for both nonvegetarians and pescatarians vs. vegans comparisons.

**Table 3.** Ten top genes with the greatest differential CpG methylation status in vegans vs. pescatarians.

Entrez Gene Name	Symbol	Fold Change	Location	Type(s)
purine rich element binding protein G	PURG	2.488	Other	other
proline rich 7, synaptic	PRR7	2.421	Plasma Membrane	other
mitochondrial ribosomal protein L19	MRPL19	2.067	Cytoplasm	other
catalase	CAT	1.864	Cytoplasm	enzyme
kinesin family member 15	KIF15	1.523	Nucleus	other
solute carrier family 38 member 6	SLC38A6	1.507	Plasma Membrane	transporter
proteasome 26S subunit, non-ATPase 5	PSMD5	1.478	Other	other
Rap associating with DIL domain	RADIL	1.433	Cytoplasm	other
amyloid beta precursor protein binding family B member 2	APBB2	1.431	Cytoplasm	other

Genes highlighted in grey are common for both nonvegetarians and pescatarians vs. vegans comparisons.

**Table 4.** Functional annotation cluster 1 report for vegans vs. nonvegetarians run with high classification stringency.

Annotation Cluster 1	Enrichment Score: 5.206834531318599								
Category	Term	Count	%	*p* Value	List Total	Fold Enrichment	Bonferroni	Benjamini	FDR
INTERPRO	IPR017970:Homeobox, conserved site	16	4.98	4.5 × 10^−7^	299	5.23	2.9 × 10^−4^	2.9 × 10^−4^	6.7 × 10^−4^
UP_KEYWORDS	Homeobox	17	5.30	3.2 × 10^−6^	317	4.21	9.5 × 10^−4^	4.7 × 10^−4^	0.004
INTERPRO	IPR001356:Homeodomain	17	5.30	4.1 × 10^−6^	299	4.12	0.003	0.001	0.006
UP_SEQ_FEATURE	DNA-binding region:Homeobox	14	4.36	9.7 × 10^−6^	313	4.70	0.011	0.011	0.016
SMART	SM00389:HOX	17	5.30	2.9 × 10^−6^	197	3.47	0.005	0.005	0.036
INTERPRO	IPR009057:Homeodomain-like	18	5.61	3.3 × 10^−5^	299	3.33	0.022	0.007	0.049

Term: Biological process. Count: Number of genes involved in this pathway. %: percentage of involved genes to the total number of genes in this analysis.

**Table 5.** Functional annotation cluster 1 report run for vegans vs. pescatarians run with high classification stringency.

Annotation Cluster 1	Enrichment Score: 1.945388640497003								
Category	Term	Count	%	*p* Value	List Total	Fold Enrichment	Bonferroni	Benjamini	FDR
UP_SEQ_FEATURE	DNA-binding region:Homeobox	8	3.76	0.0033	203	4.14	0.88	0.88	4.79
INTERPRO	IPR020479:Homeodomain, metazoa	6	2.82	0.0035	205	5.90	0.81	0.81	4.85
INTERPRO	IPR017970:Homeobox, conserved site	8	3.76	0.0051	205	3.81	0.91	0.71	7.05
UP_KEYWORDS	Homeobox	8	3.76	0.0171	208	3.02	0.99	0.52	20.09
INTERPRO	IPR001356:Homeodomain	8	3.76	0.0230	205	2.83	1.00	0.89	28.36
SMART	SM00389:HOX	8	3.76	0.0323	123	2.62	0.98	0.86	31.44
INTERPRO	IPR009057:Homeodomain-like	9	4.23	0.0327	205	2.42	1.00	0.90	37.89

**Table 6.** CpG sites identified as differentially methylated in transcription factor genes in pescatarians.

ID	Gene Name	FC in Omnivores	FC in Piscvores
BARHL1	BarH like homeobox 1(BARHL1)		−1.23555
ISL1	ISL LIM homeobox 1(ISL1)	1.23352	1.2188
NKX3–2	NK3 homeobox 2(NKX3–2)	1.25585	1.26999
NKX6–1	NK6 homeobox 1(NKX6–1)		−1.32335
EVX1	even-skipped homeobox 1(EVX1)		1.27498
HOXA2	homeobox A2(HOXA2)		1.24374
HOXC9	homeobox C9(HOXC9)		1.29757
RAX	retina and anterior neural fold homeobox(RAX)	1.27834	1.24775

Genes that are also differentially methylated in nonvegetarians are highlighted in grey.

**Table 7.** (Kyoto Encyclopedia of Genes and Genomes) Pathways enriched in genes with differential methylation in pescatarians.

Term	Count	%	*p* Value	Fold Enrichment	Bonferroni	Benjamini	FDR
hsa04390:Hippo signaling pathway	7	3.29	0.0031	4.76	0.34	0.34	3.58
hsa05014:Amyotrophic lateral sclerosis (ALS)	4	1.88	0.0120	8.21	0.80	0.55	13.16
hsa04724:Glutamatergic synapse	5	2.35	0.0235	4.50	0.96	0.65	24.25

Term: Name of KEGG pathway; Count: Number of genes involved in this pathway; %: percentage of involved genes to the total number of genes in this analysis.Pathways that were also found in pathway analysis of nonvegetarians are highlighted in grey.

**Table 8.** KEGG Pathways enriched in genes with differential methylation in nonvegetarians.

Term	Count	%	*p* Value	Fold Enrichment	Bonferroni	Benjamini	FDR
hsa05033:Nicotine addiction	5	1.56	0.0032	8.04	0.42	0.42	3.82
hsa04723:Retrograde endocannabinoid signaling	6	1.87	0.0195	3.82	0.97	0.82	21.38
hsa04921:Oxytocin signaling pathway	7	2.18	0.0279	3.00	0.99	0.80	29.23
hsa04390:Hippo signaling pathway	7	2.18	0.0287	2.98	0.99	0.71	29.94
hsa04727:GABAergic synapse	5	1.56	0.0417	3.78	1.00	0.77	40.59
hsa04810:Regulation of actin cytoskeleton	8	2.49	0.0429	2.45	1.00	0.72	41.46

Term: Name of KEGG pathway; Count: Number of genes involved in this pathway; %: percentage of involved genes to the total number of genes in this analysis.Pathways that include proteins involved in GABA and Glutamate metabolism are highlighted in grey.
